# Value Conditioning Modulates Visual Working Memory Processes

**DOI:** 10.1037/xhp0000144

**Published:** 2015-11-02

**Authors:** Paul M. J. Thomas, Lily FitzGibbon, Jane E. Raymond

**Affiliations:** 1 Bangor University; 2 School of Psychology, University of Birmingham

**Keywords:** working memory, value learning, attention, eye movements, vision

## Abstract

Learning allows the value of motivationally salient events to become associated with stimuli that predict those events. Here, we asked whether value associations could facilitate visual working memory (WM), and whether such effects would be valence dependent. Our experiment was specifically designed to isolate value-based effects on WM from value-based effects on selective attention that might be expected to bias encoding. In a simple associative learning task, participants learned to associate the color of tinted faces with gaining or losing money or neither. Tinted faces then served as memoranda in a face identity WM task for which previously learned color associations were irrelevant and no monetary outcomes were forthcoming. Memory was best for faces with gain-associated tints, poorest for faces with loss-associated tints, and average for faces with no-outcome-associated tints. Value associated with 1 item in the WM array did not modulate memory for other items in the array. Eye movements when studying faces did not depend on the valence of previously learned color associations, arguing against value-based biases being due to differential encoding. This valence-sensitive value-conditioning effect on WM appears to result from modulation of WM maintenance processes.

With experience, objects and the outcomes that follow them become associated via learning so that eventually the mere sight of an object can generate a prediction of the value of the likely consequent event. Such learned value associations influence simple visual cognitive processes even when these associations are not currently relevant. Specifically, task-irrelevant value associations can modulate spatial visual attention (Anderson, Laurent, & Yantis, 2011; Della Libera & Chelazzi, 2006, 2009; Rutherford, O’Brien, & Raymond, 2010), temporal visual attention (Raymond & O’Brien, 2009), and even the speed of perceptual processing (O’Brien & Raymond, 2012). Current views of visual working memory (WM) posit that the selective attention mechanisms that prioritize perceptual processes may also prioritize WM maintenance (Kuo, Stokes, & Nobre, 2012; Schmidt, Vogel, Woodman, & Luck, 2002; Vogel, Woodman, & Luck, 2005), suggesting that value associations might influence postencoding WM processes.

To address this possibility, we sought to provide advances over previous work investigating effects of reward association on visual WM by carefully disentangling value effects on sensory encoding from those on WM processes, per se. Not only have previous studies reported contradictory effects (Gong & Li, 2014; Infanti, Hickey, & Turatto, 2015; Wallis, Stokes, Arnold, & Nobre, 2015), their experimental designs specifically encouraged value-based selective encoding, leaving it unclear whether learned value associations can influence WM except via selective encoding. All previous studies used instrumental conditioning to imbue arbitrary stimuli with value; that is, visual orienting responses made to one stimulus (e.g., a red ring) were reinforced more than orienting to another (e.g., a green ring), thereby enhancing the likelihood of orienting to the former stimulus if encountered in the future (Thorndike’s Law of Effect; Thorndike, 1898). They then used the conditioned stimuli as memoranda in WM tasks, reporting, in some cases, better WM for the more-rewarded stimulus (Gong & Li, 2014; Wallis et al., 2015), or worse WM for no-value items that had been studied in the presence of a highly rewarded stimulus (Infanti et al., 2015). Both findings are consistent with the notion of preferential selective encoding. Furthermore, in all cases, the complex, dense memoranda arrays used were presented very briefly (≤500 ms), making selective encoding a necessity. For these reasons prior studies of value effects on WM leave open the question of whether value can modulate WM, beyond causing selective encoding.

To isolate value effects to WM itself, we designed an experiment that specifically limited the utility of value-based selective attention. The study began with an associative learning task used to imbue stimuli with value without simultaneously conditioning orienting responses that could bias selective attention during memory array exposure. During conditioning, participants selected a blank tile from an array of four to reveal a tinted face, the color of which reliably predicted a subsequent monetary outcome (gain, loss, or no outcome). Six face identities, each in four different colors (one for gain, one for loss, and two for no outcome), were presented during conditioning, allowing color-value associations to develop without making outcomes contingent on visual orienting. Later, the same tinted faces served as memoranda in a face-identity WM change-detection task. In this task, no money was gained or lost, making color-value associations irrelevant. Participants viewed a protracted study array of four faces (2000 ms), and then, after a 1000-ms blank retention interval, a single test face was presented and the participants’ task was to report whether it had been in the previous WM array. Study duration was long to reduce the impact of selective attention effects on encoding by providing time to fixate and study every face (Curby & Gauthier, 2007). To further minimize the utility of value-based selective encoding, we controlled attentional priorities by making one item perceptually salient (a color singleton) to promote its being encoded first and fully; additionally, we tested the singleton more often than any other item. The singleton was the only item in the array ever to have a gain- or loss-associated tint (although it could also have a no-outcome-associated tint); all other items had a different but common no-outcome-associated color. Of interest was whether memory for the attention-attracting color singleton depended on the value associated with its color, and whether singleton value affected WM for the remaining nonsingleton color items in the array. If value modulates WM processes, then performance for win- or loss-associated singleton faces should be different (possibly better or worse, respectively) than for singleton faces presented in a no-outcome color. We also monitored eye movements during the WM task so that we could directly assess visual orienting when the memoranda were present.

Of additional interest was whether the value associated with the singleton would have any effect on WM for the other nonsingleton, no-outcome-associated faces. If the presence of a reward-associated singleton in the study array caused a diffuse release of neuromodulators such as dopamine (Bromberg-Martin, Matsumoto, & Hikosaka, 2010; Brooks & Berns, 2013), which could in turn modulate prefrontal cortex (PFC) activity and WM more generally (Cools & D’Esposito, 2011), then WM for nonsingletons might show the same value-based effects as WM for singletons. Alternatively, if value associations modulate long-term memory (LTM) representations (Wittmann et al., 2005) and these serve to modulate WM (Ranganath, Cohen, Dam, & D’Esposito, 2004), then WM for nonsingletons (no-outcome stimuli) should be unaffected by singleton value.

## Method

### Participants

Sixteen experimentally naive adults (11 females; mean age 22.0 years; normal or corrected-to-normal vision) from the University of Birmingham completed the task for money and course credit. One participant was unable to complete because of software problems. Gaze position data were not available for two further participants, one because of technical problems and the other because of experimenter error. Data from these participants were included in the behavioral analyses, but not the gaze position analyses.

### Apparatus

A Stone SOFREP-144 computer running E-Prime 2.0 (Schneider, Eschman, & Zuccolotto, 2012) recorded data and presented stimuli on a 23-inch Asus VG278HE monitor (1920 × 1080 pixels, 60-Hz refresh) viewed from 60 cm using a chinrest. An EyeLink® 1000 desktop-mounted eye-tracker (SR Research Ltd., Ottawa, Ontario, Canada) recorded movements of the left eye with a sampling frequency of 500 Hz.

### Stimuli

**Figure 1 fig1:**
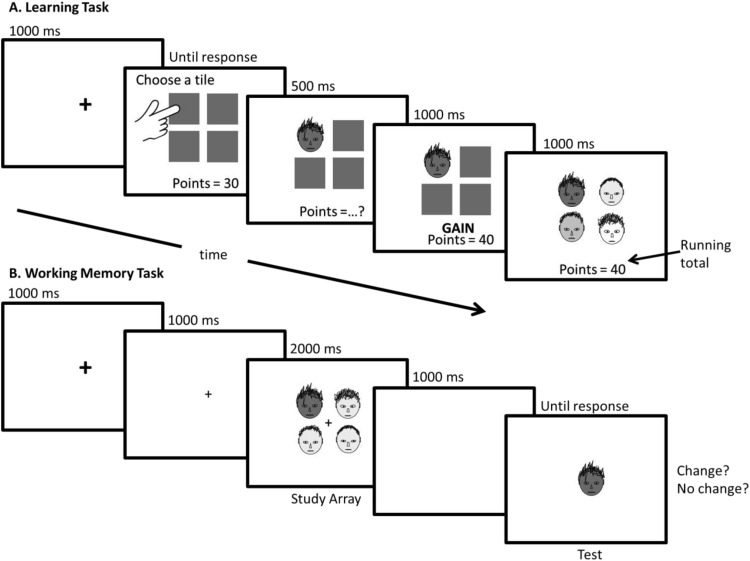
An illustration of a sample trial in the (A) value-learning task and (B) working memory (WM) task. Faces in both tasks were in fact gray-scale face photos of real people with transparent color overlays. The schematic hand shown here was not visible to participants. (A) For the learning task, four gray tiles were presented after a short fixation period. Participants chose a tile by pressing a corresponding key on the number pad. A tinted face immediately replaced the tile and a question mark replaced the running total. After 500 ms, “GAIN,” “LOSS,” or “NOTHING” (in green, red, or black, respectively) appeared and the running points total updated by +10, −10, or 0 points for win, loss, or no outcome, respectively. After 1000 ms, each remaining tile was replaced by a face with a unique tint and identity. (B) The WM task began with two successive fixation displays (each 1000 ms) showing a larger, then smaller, central cross. Then, a WM study array of four faces was presented (2000 ms), followed by a blank screen (1000 ms). All the faces in the WM array had the same color except one (singleton face). Finally, a single test face appeared centrally until the participant reported whether the identity of the test face matched one of the faces seen in the study array (no change trial) or not (change trial). The trial shown is a singleton no-change trial.

Face stimuli were grayscale bitmaps (depth 24; sized to 73 × 84 pixels; subtending 2.2° wide × 2.4° tall of visual angle) of six neutral male adults (Ekman & Friesen, 1976) overlaid with transparent color (25% opacity; yellow: R(255), G(255), B(0); magenta: R(255), G(0), B(170); green: R(0), G(255), B(0); or blue: R(0), G(51), B(255)). Color-identity combinations yielded 24 images. Tiles in the learning task (see Figure 1) were gray 2.7° squares on a white field separated by 0.3°. Faces in the WM study arrays were separated by 0.24°.[Fig-anchor fig1]

### Procedure

Participants were initially given 100 points and told to earn as many points as possible, which would later be exchanged for money (1 point = 1 penny). The session began with the learning task and ended with the WM task. Standard nine-point eye position calibration was done prior to, and at intervals of, every 40 trials during the WM task. The stimulus arrangements and details of the procedures are shown in Figure 1. In each of 48 trials of the learning task, the participant selected a tile, causing it to be replaced by a tinted face and the presentation of outcome information (+10, −10, or 0 points). Choice response time (reaction time) was recorded. Each possible identity appeared twice in each tint as the selected face, in an individually randomized order. Each color was 100% predictive of its assigned outcome (gain, loss, or nothing); these assignments were counterbalanced across participants. Participants then viewed each face (once in each tint, 24 trials) for 2500 ms, predicted an outcome (*M* = 76.9% correct, *SD* = .24), and then viewed the actual outcome, making color-outcome associations explicit.

The WM task is illustrated in Figure 1B. Each trial presented a 2000-ms study array of four faces: three in the same no-outcome associated color and one in a different (singleton) color. Each no-outcome color was equally likely to serve as the nonsingleton color. The singleton had a different no-outcome color on 50% of trials, the loss color on 25% of trials, and the gain color on remaining trials; thus each value associated color was presented equally often as the singleton. After 1000 ms, a single test face was presented centrally until response. Participants reported whether the test face’s identity matched a face in the study array or not by pressing the letter key *o* or *i*, respectively. The test face identity was equally likely to be different from all of the study array faces (no-change trial) or to match one of them (match trial), and was equally likely to have the singleton (singleton-trials) or nonsingleton (nonsingleton trial) color. Participants were made aware of these probabilities. The session comprised two blocks of 128 trials each. Each face identity was viewed an equal number of times.

### Data Analysis

WM performance was quantified using conventional *d*′ calculations for each participant and condition based on *Z*-transformed hit and false alarm probabilities. *D*′ values were analyzed using a repeated-measures anaylsis of variance (ANOVA) with value (gain/loss/no outcome) and singleton status (singleton/nonsingleton) as within-subjects factors. Planned paired-sample two-tailed *t* tests were used to compare means; α = .05.

Similar analyses were conducted on gaze position data. Gaze position was analyzed using EyeLink® Data Viewer (SR Research Ltd., Ottawa, Ontario, Canada) software. Fixations and saccades were conventionally defined (periods of pupil detection without saccade, and periods when gaze position changes were >0.1° and accelerated by >8000°/s^2^ or had a velocity of >30°/s, respectively). Four vertical rectangular (79 × 90 pixels) regions of interest (ROI) were defined for the study array display; their centers coincident with the center of each face. Mean number of fixations, total dwell time, and probability of first fixation for each ROI were determined for each participant for each study array condition.

## Results

**Figure 2 fig2:**
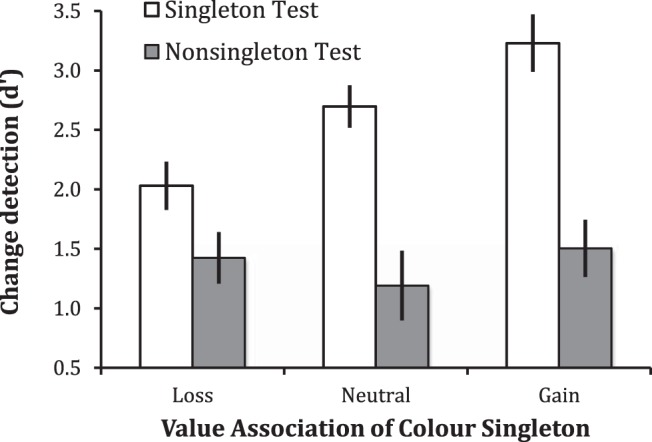
Group mean working memory performance (*d*′) when the singleton (open bars) or nonsingleton (gray bars) face was tested after viewing an array in which the color singleton had the gain-, loss-, or no-outcome-associated color. Vertical lines indicate ±1 within subject standard error.

WM performance (*d*′) depended on the value associated with the singleton presented in the array, *F*(2, 28) = 8.664, *p* = .001, η_p_^2^ = .382, and was better when a singleton versus nonsingleton was probed, *F*(1, 14) = 36.763, *p* < .001, η_p_^2^ = .724. As can be seen in Figure 2, interaction of singleton status and value was also significant, *F*(2, 28) = 7.669, *p* = .003, η_p_^2^ = .336, justifying separate ANOVAs for each status condition. Value had a significant effect in the singleton condition, *F*(2, 28) = 17.117, *p* < .001, η_p_^2^ = .550; performance was better for singleton faces seen in a gain (mean *d*′ = 3.23, *SE* = 0.23) versus no outcome (mean *d*′ = 2.70, *SE* = 0.20; *p* = .018) or loss color (mean *d*′ = 2.03, *SE* = 0.17; *p* < .001). WM for loss-colored singletons was worse than that for no-outcome singletons (*p* = .010). In contrast, singleton value had a nonsignificant effect on WM for nonsingleton faces (*F* < 1).[Fig-anchor fig2]

Singleton value had nonsignificant effects on total dwell time, number of fixations, or the probability of capturing the first fixation during WM encoding (all *F*s ≤ 1.0). Singleton status, on the other hand, had significant effects on dwell time, *F*(1, 12) = 9.316, *p* = .010, η_p_^2^ = .437, and on the probability of capturing the first fixation, *F*(1, 12) = 7.828, *p* = .016, η_p_^2^ = .395; it had a marginally significant effect on the number of fixations, *F*(1, 12) = 4.196, *p* = .063, η_p_^2^ = .259. On average, each singleton face was fixated 1.37 times per study array interval, viewed for a total of 716 ms, and attracted the first fixation on 26.8% of trials; each nonsingleton color face was fixated 1.30 times per study array, viewed for only 356 ms, and attracted the first fixation on 24.4% of trials, confirming that our experimental design successfully biased encoding in favor of the singleton. The interaction of value and status was nonsignificant for all measures (all *F*s < 1).

## Discussion

Here we show that after color-value conditioning, WM is better for stimuli with gain-associated colors and worse for stimuli with loss-associated colors, compared to that for stimuli with no-outcome associated colors, even though color-value associations were irrelevant. These effects are unlikely to result from value-based differences in selective visual attention (e.g., Anderson et al., 2011; Hickey, Chelazzi, & Theeuwes, 2010) during the study interval for the following reasons. First, the lengthy study interval we provided would have allowed plenty of time for fully encoding all the stimuli (Curby & Gauthier, 2007; Jackson & Raymond, 2008), precluding encoding limitations that were problematic for previous studies (Gong & Li, 2014; Infanti et al., 2015; Wallis et al., 2015). Second, assuming WM capacity is constrained to one or two faces (Jackson & Raymond, 2008), the experimental design would have actively biased selection for WM in favor of the critical, singleton stimulus regardless of its value association because it had greater perceptual salience and was tested more often. Furthermore, eye movements during study did not depend on the value associated with the singleton, even though a large and significant difference in WM for these conditions was found. Third, a value-based bias in selective attention during the study interval predicts that WM for nonsingleton (nonprioritized) items should have shown costs when the concurrent singleton was gain-associated (with reverse effects for loss-associated singleton conditions), an effect we did not observe. Rather, we found nonsignificant effects of value for the nonsingleton items. The current findings thus provide the first clear evidence that value associations can modulate postselection WM processes and that these effects are valenced.

Two potential mechanisms by which this could occur are (1) diffuse release of neuromodulators (e.g., dopamine) by the midbrain in response to the presentation of value-associated stimuli (Bromberg-Martin et al., 2010; Brooks & Berns, 2013) causing subsequent modulation of dopamine levels in PFC and thereby affecting WM (Cools & D’Esposito, 2011); and (2) differential value-related support to WM maintenance from LTM (Ranganath et al., 2004). The neuromodulation account would have been supported had we found evidence for a scene-wide effect on WM of the value associated with the singleton in the WM array, but this was not found. In contrast, clear support for the LTM notion is provided by the large and significant effect of singleton value association on WM for this item without a concurrent effect on other items. An LTM account of value-based WM modulation is inspired by evidence that LTM appears to support WM maintenance (Ranganath et al., 2004) and that LTM is better for stimuli that predict rewards than for those that do not (Wittmann et al., 2005). The LTM account can potentially explain why value conditioning of a feature (in this case, color) was able to modulate WM for an unrelated feature of the same object (face identity), even when the value-associated feature was irrelevant to the WM task. Perhaps LTM representations established for each color-face conjunction were particularly strong for gain-associated colors and weak for loss-associated colors, and thus helped or hindered WM maintenance, respectively. Whatever the mechanism, we show here that WM can be boosted by reward associations and diminished by loss associations, an effect that may serve to maintain reward-directed behavior in healthy humans.

## References

[c1] Anderson B. A., Laurent P. A., & Yantis S. (2011). Value-driven attentional capture. Proceedings of the National Academy of Sciences, USA, 108, 10367–10371. 10.1073/pnas.1104047108 PMC3121816 21646524

[c3] Bromberg-Martin E. S., Matsumoto M., & Hikosaka O. (2010). Dopamine in motivational control: Rewarding, aversive, and alerting. Neuron, 68, 815–834. 10.1016/j.neuron.2010.11.022 21144997 PMC3032992

[c4] Brooks A. M., & Berns G. S. (2013). Aversive stimuli and loss in the mesocorticolimbic dopamine system. Trends in Cognitive Sciences, 17, 281–286. 10.1016/j.tics.2013.04.001 23623264

[c5] Cools R., & D’Esposito M. (2011). Inverted-U-shaped dopamine actions on human working memory and cognitive control. Biological Psychiatry, 69(12), e113–e125. 10.1016/j.biopsych.2011.03.028 21531388 PMC3111448

[c6] Curby K. M., & Gauthier I. (2007). A visual short-term memory advantage for faces. Psychonomic Bulletin & Review, 14, 620–628. 10.3758/BF03196811 17972723

[c7] Della Libera C., & Chelazzi L. (2006). Visual selective attention and the effects of monetary rewards. Psychological Science, 17, 222–227. 10.1111/j.1467-9280.2006.01689.x 16507062

[c8] Della Libera C., & Chelazzi L. (2009). Learning to attend and to ignore is a matter of gains and losses. Psychological Science, 20, 778–784. 10.1111/j.1467-9280.2009.02360.x 19422618

[c9] Ekman P., & Friesen W. V. (1976). Pictures of facial affect. Palo Alto, CA: Consulting Psychologists Press.

[c10] Gong M., & Li S. (2014). Learned reward association improves visual working memory. Journal of Experimental Psychology: Human Perception and Performance, 40, 841–856. 10.1037/a0035131 24392741

[c11] Hickey C., Chelazzi L., & Theeuwes J. (2010). Reward changes salience in human vision via the anterior cingulate. Journal of Neuroscience, 30, 11096–11103. 10.1523/JNEUROSCI.1026-10.2010 20720117 PMC6633486

[c12] Infanti E., Hickey C., & Turatto M. (2015). Reward associations impact both iconic and visual working memory. Vision Research, 107, 22–29. 10.1016/j.visres.2014.11.008 25481632

[c13] Jackson M. C., & Raymond J. E. (2008). Familiarity enhances visual working memory for faces. Journal of Experimental Psychology: Human Perception and Performance, 34, 556–568. 10.1037/0096-1523.34.3.556 18505323 PMC4262787

[c14] Kuo B.-C., Stokes M. G., & Nobre A. C. (2012). Attention modulates maintenance of representations in visual short-term memory. Journal of Cognitive Neuroscience, 24, 51–60. 21736457 10.1162/jocn_a_00087 PMC3480577

[c15] O’Brien J. L., & Raymond J. E. (2012). Learned predictiveness speeds visual processing. Psychological Science, 23, 359–363. 10.1177/0956797611429800 22399415

[c16] Ranganath C., Cohen M. X., Dam C., & D’Esposito M. (2004). Inferior temporal, prefrontal, and hippocampal contributions to visual working memory maintenance and associative memory retrieval. Journal of Neuroscience, 24, 3917–3925. 10.1523/JNEUROSCI.5053-03.2004 15102907 PMC6729418

[c17] Raymond J. E., & O’Brien J. L. (2009). Selective visual attention and motivation: The consequences of value learning in an attentional blink task. Psychological Science, 20, 981–988. 10.1111/j.1467-9280.2009.02391.x 19549080

[c18] Rutherford H. J. V., O’Brien J. L., & Raymond J. E. (2010). Value associations of irrelevant stimuli modify rapid visual orienting. Psychonomic Bulletin & Review, 17, 536–542. 10.3758/PBR.17.4.536 20702874

[c19] Schmidt B. K., Vogel E. K., Woodman G. F., & Luck S. J. (2002). Voluntary and automatic attentional control of visual working memory. Perception & Psychophysics, 64, 754–763. 10.3758/BF03194742 12201334

[c20] Schneider W., Eschman A., & Zuccolotto A. (2012). E-Prime user’s guide. Pittsburgh, PA: Psychology Software Tools.

[c21] Thorndike E. L. (1898). Animal intelligence: An experimental study of the associative processes in animals. Psychological Review: Psychological Monographs, 8, 1–109.

[c22] Vogel E. K., Woodman G. F., & Luck S. J. (2005). Pushing around the locus of selection: Evidence for the flexible-selection hypothesis. Journal of Cognitive Neuroscience, 17, 1907–1922. 16356328 10.1162/089892905775008599

[c23] Wallis G., Stokes M. G., Arnold C., & Nobre A. C. (2015). Reward boosts working memory encoding over a brief temporal window. Visual Cognition, 23(1–2), 1–22.

[c25] Wittmann B. C., Schott B. H., Guderian S., Frey J. U., Heinze H.-J., & Düzel E. (2005). Reward-related FMRI activation of dopaminergic midbrain is associated with enhanced hippocampus-dependent long-term memory formation. Neuron, 45, 459–467. 10.1016/j.neuron.2005.01.010 15694331

